# Analysis of the prevalence, risk factors, and clinical characteristics of osteoporosis in patients with essential hypertension

**DOI:** 10.1186/s12902-022-01080-w

**Published:** 2022-06-27

**Authors:** Hai-Long Wu, Jie Yang, Yu-Chi Wei, Jian-Yu Wang, Yu-Yan Jia, Luan Li, Lu Zhang, Yan Lu, Zong-Jian Luo, Xiang-Yang Leng

**Affiliations:** 1 Changchun University of Chinese Medicine, Changchun, Jilin, China; 2 Changchun Medical College, Changchun, Jilin, China; 3 The Affiliated Hospital to Changchun University of Chinese Medicine, Changchun, Jilin, China

**Keywords:** Antihypertensive medication, Creatinine, Essential hypertension, Influencing factors, Osteoporosis

## Abstract

**Background:**

The present study investigated the prevalence of osteoporosis (OP) among patients with essential hypertension (EH) in the Changchun community and analysed the correlation between EH and OP.

**Methods:**

The study included 425 subjects with EH and 425 age- and sex-matched healthy controls. Bone mineral density (BMD) and serum creatinine (CR) levels were measured, and the subjects' current EH and OP statuses were surveyed to analyse the correlation between EH and OP.

**Results:**

The EH group exhibited lower BMD and a higher rate of having OP than the control group, and this difference was statistically significant (*p* < 0.05). A significant sex difference in the BMD T-score was observed among the subjects (male: − 1.19 ± 1.55, female: − 1.70 ± 1.34). In both the EH group and the control group, the rate of having OP in females was greater than that in males. However, the OP prevalence among subjects with EH varied significantly by age, body weight, fracture history, nocturnal urination frequency, depression and anxiety status, duration of hypertension, and antihypertensive medication use (*p* < 0.05). Two-way analysis of variance suggested an effect of the interaction between different EH statuses and bone mass conditions on the serum CR values (F = 3.584, *p* = 0.028, bias η^2^ = 0.008).

**Conclusions:**

The prevalence of OP and low BMD were significantly higher among subjects with EH than among healthy controls. Additionally, the findings indicate that age, weight, fracture history, nocturnal urination frequency, depression and anxiety, duration of hypertension and antihypertensive drug use may be correlated to having OP in EH subjects, requiring further studies. Moreover, serum CR levels in subjects with different bone mass profiles were strongly influenced by the presence or absence of EH, and the serum CR levels differed significantly with the interaction of these two factors.

## Background

Osteoporosis (OP) and essential hypertension (EH) are common in both the middle-aged and elderly populations and often occur as comorbid diseases [1]. With increasing age and life expectancy, the prevalence rates of OP and EH, either alone or in combination, have increased considerably [2]. According to a 2019 study [3], the prevalence of reduced bone mineral density (BMD) is significantly higher among patients with EH than among healthy individuals. Additionally, hypertension treatment affects BMD and exacerbates OP [4]. In the long term, EH patients are more likely to develop OP [5]. EH and OP share not only several factors [6], but also a similar aetiology [7]. OP is correlated with the pathogenesis of hypertension [8]. In the clinic, treatment to control blood pressure is necessary for EH patients, but the drugs used to control blood pressure contain components that can adversely affect bone quality; for example, efonidipine can interact with calcium ions [9]. Therefore, we investigated whether the risk factors for EH, such as the use of different types of antihypertensive drugs, can aggravate or reduce the occurrence of OP. The findings of this study can help in determining whether it is possible to prevent antihypertensive drugs from having an impact on bone quality when administered to people diagnosed with EH who also have OP risk factors. In this study, a clinical epidemiological investigation was conducted based on this connection, and the variables associated with EH and OP were preliminarily explored through statistical methods, providing a reference for future research on risk factors for OP among EH patients.

## Methods

### Subject selection criteria

The present study included 425 subjects between 50 and 80 years old who were diagnosed with EH and had at least a 2-year duration of chronic disease. The control group comprised an equal number of healthy individuals in the same age group. We used questionnaires to recruit hypertensive subjects, as they have a better awareness of whether they already have hypertension. In addition, we screened patients with a history of hypertension for at least 2 years and ascertained their hypertension and medication status through an interview, which provided basic data support for subsequent analysis of the correlation between EH and OP. The control group comprised an equal number of non-EH subjects with the same sex and age distribution. Finally, 1786 subjects were screened, of whom only 425 could be included as controls. In addition, we ensured that the sex and age distribution was exactly the same between the EH and control groups.

Subjects with the following conditions were excluded: pituitary, thyroid, parathyroid, adrenal, or gonadal diseases or tumours; severe heart, liver, kidney, central nervous system or psychiatric diseases; type I or type II diabetes or chronic metabolic disorders other than OP; dependence on drugs other than antihypertensive medication during the study period; spinal idiopathic disease; and other major medical conditions, such as infection or cancer. Patients who had received anti-osteoporosis treatment and pregnant or lactating women were also excluded from the study.

The studied population comprised residents of the local community in the Changchun urban area who were recruited from October 2017 to December 2017. Cluster sampling was performed to include a diverse sample of subjects in the community who met all the inclusion criteria. The general information, sociodemographic data, and data on the medical status of all the participants were collected through a questionnaire survey. The detailed medical history of the participants was recorded, and physical examination and laboratory tests were conducted to exclude subjects with abnormal results or certain medical conditions. The study procedures were explained in detail to the participants, and signed informed consent forms were obtained from all the participants. All procedures were conducted in accordance with the relevant guidelines and regulations. The Institutional Review Board of Changchun University of Traditional Chinese Medicine Hospital approved the study.

### Representational considerations

The subjects of this study are all from Luyuan District and Chaoyang District of Changchun City, both of which are old urban areas that cover a wide range of risk exposures. Therefore, the representativeness of the sample was improved, and the systematic error that occurs in the selection process of research subjects is reduced.

BMI was calculated based on weight and height, and related studies [10–12] have shown that simple obesity and overweight are protective factors against osteoporosis but are risk factors for EH. Therefore, in this study, weight was selected as one of the independent indicators. In addition, The estimated glomerular filtration rate (eGFR) is a classic indicator used to evaluate renal function, and the reasons for including the serum creatinine (CR) level as an indicator in this study are as follows: 1. The serum creatinine level has a positive correlation with the patients’ hypertension state [13], and 2. the serum creatinine level has a negative correlation with OP development [14]. Therefore, we included the serum creatinine level in the present study as the link between EH and OP.

### BMD assay and demographic characteristics of patients and controls

BMD levels in the lumbar spine and left hip were determined through dual energy X-ray absorptiometry (DXA) by using a Discovery Wi (S/N 88,317), produced by American Hologic. According to the World Health Organization website (http://slideplayer.com/slide/4493823/) that provides the relationship between the BMD measurement value (T value) and OP, the subjects were classified into the following groups: normal bone mass (T value ≥  − 1.0), osteopenia (− 2.5 < T value <  − 1.0), and osteoporosis (T value ≤  − 2.5) [15]. The anthropometric variables weight (kg) and height (cm) were measured in a standardized manner.

In this study, specific professionals who were designated to perform BMD testing conducted strict quality control every day before testing subjects. After completing the diagnostic tests for some functional indicators and scanning the lumbar vertebral phantom provided by the manufacturer, the diagnostic report was evaluated. If a diagnostic report is not generated, the test should be performed a second time. In the case of two consecutive failures or if the difference between the two consecutive scans and the baseline value is greater than 1.5%, the researcher must contact the manufacturer to identify the cause. In addition, if a deviation from the baseline trend is observed in the quality control chart, the inspector should check whether it is a calibration drift. The height and weight of the subjects must be measured before the bone density measurement; in addition, the date of birth (year/month/day) must be collected to obtain more accurate measurement results. Furthermore, any accessories worn by patients, such as watches, bracelets, and rings, should also be removed while measurements are taken.

### Data processing and statistical analysis

EpiData 2.1b was used to establish the database, and SPSS (version 26.0) 10 was used for statistical processing with a two-sided p value of 0.05. The observations in the present study were independent of each other. No significant outlier was present in the observed variables, which were nearly normally distributed within each group and exhibited equal variance. The following tests were selected according to the type of variable and the need for clinical analysis:

- (1) Independent sample t tests were used to analyse group differences in age and body weight between the EH group and the control group.
- (2) The Mann–Whitney *U* test was used to analyse differences in the BMD T-score and CR levels between the EH group and the control group.
- (3) For the count data, Pearson’s correlation coefficient and the chi-square test were used if the expected frequency of all cells was > 5, whereas Fisher's exact test was used if the expected frequency was < 5. Analysis of covariance was used to control for the effects of confounding variables, and Bonferroni correction was applied to adjust for multiple tests.
- (4) The chi-square test was used to analyse differences between the EH group and the control group in terms of sex and bone mass. The clinical correlations of sex, age, fracture history, nocturnal urination frequency, depression and anxiety status, duration of EH, and antihypertensive drug use with bone mass status were also analysed within the EH group. Additionally, the column proportions were compared, and the p values were adjusted (through the Bonferroni method) in the Z-test to compare between-group differences in the demographic and clinical variables according to different bone mass profiles and intragroup differences (within the EH group) in nocturnal urination frequency, depression and anxiety status, duration of EH, and antihypertensive medication use in a two-by-two comparison.
- (5) The Mantel–Haenszel chi-square test was used to determine whether there was a linear correlation between age and the rate of OP within the EH group, and the strength of the association was determined according to Pearson’s correlation coefficient *R*.
- (6) Two-factor analysis of variance (ANOVA) was used to evaluate the effect of different EH statuses and bone mass conditions on CR. Box plots were used to test for outliers, the Shapiro–Wilk test was used to determine the normality of the data distributions, and Levene’s chi-square test was used to determine isotropy.

## Results

### Demographic characteristics of the patients and controls

The effects of confounding factors such as sex and age were excluded from the present study. Table 1 summarizes the demographic characteristics of the patients and the controls.

**Table 1:** Demographic characteristics of the EH group and the control group

	**EH group (*N* = 425)**	**Control group** **(*N* = 425)**	**Test value**	***p***
**Age (years)**	64.60 ± 6.70	63.96 ± 7.00	T = 1.358	0.173
**Sex**			χ^2^ = 0.000	1.000
Male	112 (30.59%)	112 (27.65%)		
Female	313 (69.41%)	313 (72.35%)		
**BMD**			χ^2^ = 12.968	0.002
Normal	119_a_ (28.0%)	167_b_ (39.3%)		
Osteopenia	177_a_ (41.6%)	159_a_ (37.4%)		
Osteoporosis	129_a_ (30.4%)	99_b_ (23.3%)		
**Lumbar spine T-score**	-1.54 ± 1.43	-1.34 ± 1.45	U = 83,167.000	0.046
**Body weight (kg)**	66.15 ± 10.11	62.23 ± 9.21	T = 5.760	< 0.001
**CR (µmol/L)**	70.68 ± 0.88	69.69 ± 0.72	U = 90,610.000	0.934
**eGFR (mL/min/1.73 m**^**2**^**)**			χ^2^ = 6.412	0.011
≥ 60	396 (93.18%)	412 (96.94%0		
< 60	29 (6.82%)	13 (3.06%)		

Each subscript letter indicates a subset of the EH group or control group. At the 0.05 level, there is no significant difference between the column ratios of these categories

### OP prevalence in patients and controls

**Fig. 1:**
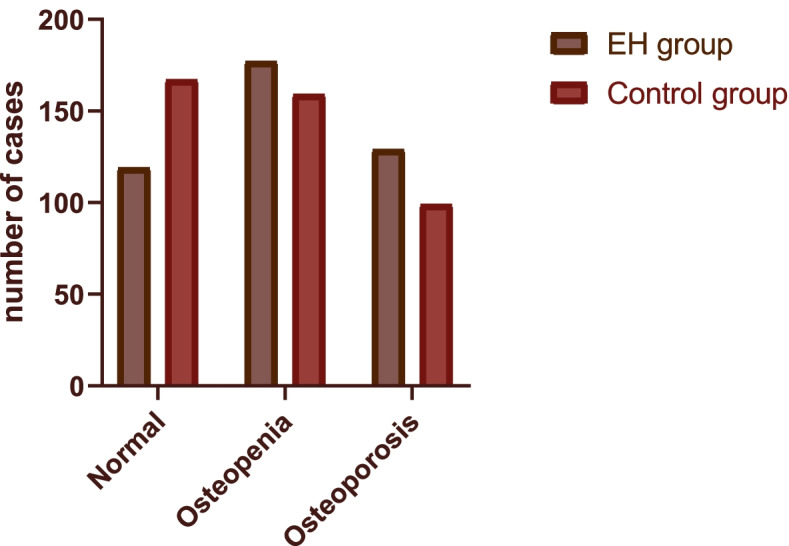
Numbers of participants in the three categories of BMD in the EH group and the control group

The prevalence of OP in the EH group was significantly higher than that in the control group (χ^2^ = 12.968; *p* = 0.002) (Table 1). Figure 1 shows the numbers of subjects in the three categories of BMD in the EH group and the control group. In both the EH group and the control group, the rate of having OP in females was greater than that in males. Correspondingly, a significant sex difference in the BMD T-score was observed among the subjects (male: − 1.19 ± 1.55, female: − 1.70 ± 1.34). Further analysis showed that although the body weight of the subjects with EH was significantly higher than that of the healthy controls (*p* < 0.001), the CR levels between the two groups did not vary significantly (*p* = 0.934).

### Demographic and clinical variables in the non-OP and OP subgroups of the EH group

A comparison of the OP and non-OP subgroups in the EH group indicated the OP prevalence (Table 2). Table 2 shows that age and OP prevalence exhibited a linear correlation (χ^2^ = 8.991, *p* = 0.001), and the occurrence of OP increased with age (*R* = 0.146 and *p* = 0.003). Additionally, the body weight of the non-OP subgroup was approximately similar to that of the OP subgroup (t = 0.874, *p* = 0.383). However, the OP prevalence were higher in subjects with previous fractures (χ^2^ = 3.934, *p* = 0.047). Other factors identified to be related to OP were nocturnal urination frequency, depression and anxiety status, duration of hypertension, and antihypertensive medication use. The percentage of EH subjects who never woke up at night to urinate, who were not depressed and anxious was lower in the OP subgroup than in the non-OP subgroup. Based on the duration of EH, the proportion of EH subjects with 6–10 years of illness or more was higher in the OP subgroup (Table 2).

**Table 2:** Demographic and clinical characteristics of patients with EH with and without OP

	**Non-OP subgroup (*N* = 296)**	**OP subgroup (*N* = 129)**	**Test value**	***p***
**Sex**			χ^2^ = 0.228	0.633
Male	80 (27.0%)	32 (24.8%)		
Female	216 (73.0%)	97 (75.2%)		
**Age (years)**			χ^2^ = 9.015	0.011
50–59	69_a_ (23.3%)	17_b_ (13.2%)		
60–69	154_a_ (52.0%)	65_a_ (50.4%)		
70–79	73_a_ (24.7%)	47_b_ (36.4%)		
**Body weight (kg)**	65.86 ± 9.84	66.80 ± 10.71	U = 19,583.000	0.673
**Fracture history**			χ^2^ = 3.934	0.047
None	237 (80.1%)	92 (71.3%)		
Yes	59 (19.9%)	37 (28.7%)		
**Nocturnal urination frequency**			χ^2^ = 7.461	0.024
None	82_a_ (27.7%)	20_b_ (15.5%)		
1–2 times	159_a_ (53.7%)	79_a_ (61.2%)		
3 or more	55_a_ (18.6%)	30_a_ (23.3%)		
**Depression/anxiety status**			χ^2^ = 8.751	0.013
None	231_a_ (78.3%)	84_b_ (65.1%)		
Mild	57_a_ (19.3%)	38_a_ (29.5%)		
Moderate	7_a_ (2.4%)	7_a_ (5.4%)		
**Course of hypertension**			χ^2^ = 12.669	0.005
2–5 years	126_a_ (42.6%)	32_b_ (24.8%)		
6–10 years	88_a_ (29.7%)	49_a_ (38.0%)		
11–19 years	31_a_ (10.5%)	21_a_ (16.3%)		
more than 20 years	51_a_ (17.2%)	27_a_ (20.9%)		
**Drug combination**			χ^2^ = 4.770	0.029
Only one	264 (89.2%)	105 (81.4%)		
2 or 3	32 (10.8%)	24 (18.6%)		
**Types of Antihypertensive medication use**			χ2= 36.722	< 0.001
Irregular use	13 (4.4%)	17 (13.2%)		
Amlodipine	125 (42.2%)	77 (59.7%)		
Nifedipine	12 (4.1%)	8 (6.2%)		
Propranolol	27 (9.1%)	5 (3.9%)		
Captopril	26 (8.8%)	5 (3.9%)		
Valsartan	71 (24.0%)	9 (7.0%)		
Proprietary Chinese Medicine (pCM)	22 (7.4%)	8 (6.2%)		
**CR (µmol/L)**	73.95 ± 18.11	69.35 ± 17.05	U = 15,489.000	0.002
**eGFR** **(mL/min/1.73 m**^**2**^**)**			χ^2^ = 0.007	0.934
≥ 60	276 (93.24%)	120 (93.02%)		
< 60	20 (6.76%)	9 (6.98%)		

Each subscript letter indicates a subset of the non-OP subgroup or OP subgroup. At the 0.05 level, there is no significant difference between the column ratios of these categories

### OP in subjects treated with different antihypertensive medications

**Fig. 2:**
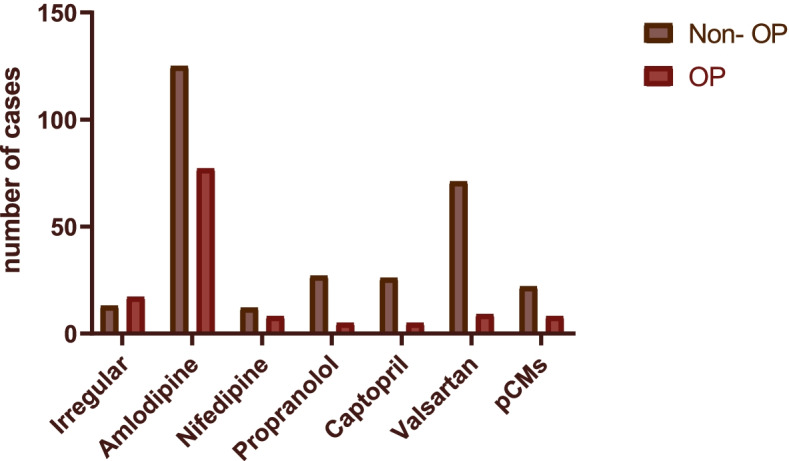
Numbers of EH subjects with OP across different antihypertensive drugs. The EH subjects were divided into 7 groups based on the type of antihypertensive medication used. The difference in the numbers of subjects with OP across the 7 groups was statistically significant (χ^2^ = 36.722, *p* < 0.001)

As shown in Table 2, among all subjects receiving a combination of two or three antihypertensive drugs, the proportion in the OP subgroup was much higher than that in the non-OP subgroup. The present study demonstrated that treatment with a combination of antihypertensive drugs increased the prevalence of OP among EH subjects compared with treatment with only one type of antihypertensive drug (χ^2^ = 4.770, df = 1, *p* = 0.029). Furthermore, Fig. 2, which shows the numbers of EH subjects with OP across different types of antihypertensive drugs, indicates that prolonged use of BP medications and multiple BP medications were associated with increased prevalence of OP (χ^2^ = 36.722, *p* < 0.001).

### Effect of differences in EH and OP prevalence on CR

No significant difference was observed in the CR levels between the EH and control groups (Table 1); however, the CR concentrations were significantly higher in the non-OP subgroup than in the OP subgroup within the EH group (*U* = 15,489.000, *p* = 0.002). An effect of the interaction between EH status and bone mass condition on the CR level was observed (F = 3.584, *p* = 0.028, bias η^2^ = 0.008). The separate effect analysis suggested that the effect on CR values differed according to the different bone mass conditions and the presence or absence of EH. Among the EH study subjects, significant differences in CR expression were observed according to the different bone mass conditions (F = 6.346, *p* = 0.002, bias η^2^ = 0.015). Additionally, serum CR levels in the study subjects with different bone mass conditions were more strongly affected by the presence or absence of EH. The CR levels varied significantly under the interaction of EH and OP, and use of the CR concentration may seriously reduce the diagnostic efficacy of OP in subjects with EH.

## Discussion

EH and OP are two major age-related diseases [16] that contribute to considerable morbidity and mortality in the middle-aged and elderly populations by inducing cardiovascular disease, fragility fractures, and associated complications and sequelae [17]. Both EH and OP are multifactorial diseases in which factors such as genetics and lifestyle contribute to the pathogenesis. The prevalence rates of EH and OP increase with increasing life expectancy. Studies have demonstrated that EH and OP share common risk factors and similar pathological mechanisms [18]. N. Hijazi et al. [19] asserted that there is no connection between EH and OP, while H. Poudyal et al. [20] reported that the two are connected. The inclusion criteria for patients with hypertension in the study by H. Poudyal et al. were a blood pressure ≥ 130/85 mmHg or a history of hypertension medication use. Our study included people who had hypertension for at least two years. From a clinical point of view, this group of subjects regularly used medication to control blood pressure. Additionally, because the change in BMD in patients with OP is a long-term process, we set the history of hypertension to 2 years. This time period is adequate for analysing the correlation between EH and BMD changes, which can be analysed as a related variable. In this research, we confirmed that there is a certain connection between the two factors. The present study revealed a significantly higher prevalence of OP in the EH group than in the control group, suggesting that EH may increase OP prevalence. This finding is consistent with that of other studies [18]. This study also identified several factors that were significantly associated with the occurrence of OP in EH patients, including sex, age, history of fractures, CR, frequency of nocturnal urination, depression and anxiety, duration of hypertension, and use of antihypertensive drugs. It is suggested that the co-occurrence of EH and OP may accelerate disease progression, thus forming a vicious cycle.

The mechanism underlying the occurrence of OP in patients with HTN is not fully understood. However, the renin-angiotensin–aldosterone system (RAAS) is known to play a crucial role in blood pressure regulation and fluid homeostasis [6]. The role of the RAAS in OP has also been reported in experimental studies, where osteoblasts were found to exhibit components of the RAAS, including angiotensin 1 receptor (AT1R), angiotensin 2 receptor (AT2R), and aldosterone receptor. In an experimental study [15], RAAS activation or chronic angiotensin II (Ang II) injection increased bone resorption, whereas a lack of AT1R was associated with an increase in bone strength. Ang II can also induce mitochondrial oxidative stress and damage mesenchymal-derived osteoblasts by reducing sirtuin 1 (*SIRT*1) expression. In experimental models [16], *SIRT1* expression was found to be positively associated with bone mass. In patients with EH, the use of RAAS inhibitors, including angiotensin-converting enzyme (ACE) inhibitors, (for 2 years or less) was associated with considerable rates of fracture development; however, their long-term use was associated with a reduced fracture risk [9, 15, 17] , which is consistent with the results of the current study. Additionally, the present study demonstrated that the use of calcium channel-blocking drugs increased the OP prevalence in subjects with EH, which may be related to the inhibition of the inward flow of extracellular Ca^2+^ by this class of drug. Because antihypertensive medications are widely used with elderly patients and EH and OP are often comorbid, the effects of antihypertensive therapy on bone and fracture rates should be considered prior to its clinical application.

The present study focused on body weight and CR levels. On the one hand, body weight and CR levels show a complex trend under the influence of both diseases. The impact of obesity on the odds of EH development may exceed its protective impact on skeletal health in middle-aged and elderly individuals [18]. Although weight gain may reduce the OP prevalence to some extent, this reduction is quite limited, and the rates of EH may be significantly increased. On the other hand, weight loss may be associated with reduced rates of hypertension and no considerable increase in OP prevalence. A related study [21] demonstrated that the addition of renal function measurements as a risk factor for fracture did not improve the OP risk factors (e.g., age, weight, and hip BMD) in postmenopausal women without moderate or severe coronary artery disease. Additionally, the present study provides circumstantial evidence that although increasing CR concentrations may improve the prediction of fracture rates to some extent, use of the CR concentration may seriously reduce the diagnostic efficacy of OP in generally asymptomatic middle-aged and elderly adults with EH.

The present study has certain limitations. First, although there are many factors that can jointly influence OP and EH, due to limited data, this study could adjust for only a limited number of confounders and did not use propensity score matching to better eliminate them. Second, our cohort was relatively young for the OP study. We should allow more time to recruit a larger number of subjects, especially in for the control group, as it is very difficult to find older individuals free of either EH or OP. Third, the study was an analytical cross-sectional study, so it is difficult to establish causality. The existing data are also insufficient to determine the association of some hypertension (HTN) drugs with OP. Although we tried our best to obtain objective data, some bias still existed in the individual lifestyle data. Therefore, based on the present study, prospective cohort studies should be considered for further research in this domain.

## Conclusions

Both EH and OP have become common in the elderly population, and EH may be a strong causative factor for OP. The extent to which the shared factors for these diseases affect bone mass status warrants further definitive quantitative analysis in the future. Therefore, we recommend that along with effective screening for hypertension, bone density testing should be promoted in the elderly population, especially for patients diagnosed with hypertension, for early screening and early intervention. Considering the closely related manifestations of these two diseases, proper blood pressure control and rational use of medications in the middle-aged and elderly populations may improve bone health and prevent the occurrence of fractures in patients with hypertension.

## Data Availability

The datasets used and/or analyzed during the current study available from the corresponding author on reasonable request.
